# Highly Cited Artificial Intelligence Research Studies Published in Neurosurgical Journals: A Bibliometric Analysis

**DOI:** 10.7759/cureus.98191

**Published:** 2025-11-30

**Authors:** Yazid Maghrabi, Abdulhakim B Jamjoom, Abdulhadi Algahtani, Ohood H Alshareef, Omar M Jamjoom, Moajeb Alzahrani

**Affiliations:** 1 Neurosurgery, King Abdulaziz Medical City, Jeddah, SAU; 2 Medicine, King Abdullah International Medical Research Center, Jeddah, SAU; 3 College of Medicine, King Saud Bin Abdulaziz University for Health Sciences, Jeddah, SAU; 4 Neurosurgery, King Saud Bin Abdulaziz University for Health Sciences, Jeddah, SAU; 5 Neurological Surgery, College of Medicine, King Saud Bin Abdulaziz University for Health Sciences, Jeddah, SAU; 6 Pharmaceutical Care Services, King Abdulaziz Medical City, Jeddah, SAU; 7 Pharmaceutical Care Services, King Saud Bin Abdulaziz University for Health Sciences, Jeddah, SAU

**Keywords:** ai, ai and machine learning, artificial intelligence, neurosurgery, virtual reality (vr)

## Abstract

Neurosurgery is inherently suited for artificial intelligence (AI) integration due to its reliance on advanced technologies. Although AI research is expanding in neurosurgery, citation patterns of impactful publications remain underexplored. We identified AI-focused articles published in neurosurgical journals (NSJs) with ≥100 citations using PubMed and Google Scholar. A total of 66 articles were analyzed. Data collected included article age, country of first author, journal, article type, subspecialty, AI technology used, and application. Citation analysis was performed using mean difference testing across subgroups. The median article age was 8.5 years (range: 1-30 years). Most articles originated from the United States (34, 52%) and were published in Neurosurgery (23, 35%). Technical notes (26, 39%) and review articles (23, 35%) were the dominant formats. General neurosurgery and spine were the leading subspecialties (each 21, 32%). AI technologies included virtual reality (VR) (19, 29%), machine learning (ML) (13, 20%), and augmented reality (10, 15%). The primary applications were surgical planning/assistance (33, 50%) and training (12, 18%). Median citations per article were 173 (range: 100-406). Higher citation rates correlated with review articles, recent publication (within 10 years), and a general neurosurgery focus. AI type and application had no significant impact on citation count. Highly cited AI research in neurosurgery predominantly originates from the United States, focuses on general neurosurgery and spine, and employs VR and ML for planning and training. Citation impact is driven more by study type and recency than by AI modality. Continued original research is vital to integrate AI advancements into standard neurosurgical practice.

## Introduction and background

Artificial intelligence (AI) is a general term used to describe machines and computers performing tasks usually requiring human intelligence [1-4]. The use of AI in the various medical disciplines remains a subject of considerable interest. Over the last two decades, an extensive body of AI-related literature covering a wide range of specialties, practices, and technologies has become available [5-8]. Neurosurgery is a field of clinical medicine that generates a large amount of data due to the routine use of high-tech medical equipment and medical information systems. These factors predispose the specialty to the adoption of AI technologies [9-11]. The core subfields of AI in neurosurgery include medical robotics and surgical technology, computer vision, machine learning (ML), deep learning (DL), and natural language processing (NLP) [5,10,12,13].

Bibliometric analysis is a quantitative research method used to evaluate the academic impact, productivity, and influence of publications within a specific field. It systematically examines publication patterns, citation metrics, collaboration networks, and thematic evolution to identify the most influential research trends and knowledge gaps in a discipline [7].

Medical robotics and surgical technology refer to the utilization of advanced technological systems in surgical procedures to assist or automate parts of the surgery to increase precision, reduce human error, and enhance recovery [5,9,10]. This broader category encompasses technologies such as virtual reality (VR), augmented reality (AR), neuronavigation, and robotics [5,14,15]. VR technology entails complete submersion into a virtual environment facilitated by specialized equipment. It is used in surgical training, simulation, and planning, allowing the surgeon to practice procedures before performing them on real patients [5,6,10]. AR technology overlays digital information such as images, sounds, or data into the real world in real-time.

It also helps surgeons to visualize anatomical structures during procedures and aid in medical training [5,6,15]. Neuronavigation technology is a computer-assisted system that uses preoperative images such as MRI or CT, combined with intraoperative tracking to provide real-time, 3D guidance for surgeons that helps them to precisely navigate and target specific areas of the brain or spine, thus enhancing accuracy and reducing risks in complex procedures [5,15].

Robotics technology involves the use of robotic systems to assist surgeons in performing highly precise and minimally invasive procedures [5,14]. Computer vision technology involves the use of AI algorithms to analyze and interpret medical images, such as MRI and CT scans, in real time during surgery. It enhances the surgeon’s ability to identify and navigate critical structures [12,16]. ML is a subset of AI concerned with the use of data to create self-teaching and self-improving algorithms. By leveraging large datasets, ML applications can enhance the decision-making process [13,15,16]. DL, a subset of ML, uses artificial neural networks (ANNs) to automatically extract, analyze, and grasp pertinent information from large datasets [13,15,16]. These deep neural networks are particularly effective for tasks such as image and speech recognition, NLP, and autonomous systems.

In neurosurgery, the combination of AI, ML, and DL has the potential to improve clinical treatment, diagnostic, and prognostic outcomes, and assist neurosurgeons in making decisions during surgical interventions. NLP allows for the extraction of meaningful clinical insights from unstructured data in electronic health records. It supports the development of clinical decision support systems that analyze medical literature and guidelines, offering personalized treatment recommendations for complex cases [10,17].

The citation number of an article might not be a criterion for quality assessment; nevertheless, publications with higher citation numbers are considered a milestone in any field and can affect the research and clinical approach [18,19]. It is also recognized that an article’s citation number will affect the publishing journal’s impact factor (IF) and can be regarded as reflective of the article's endorsement, efficacy, quality, and the author’s reputation [18,19]. Citation analysis allows researchers to identify the most cited publications in their field. Assessment of the most influential publications in any subject will enhance knowledge of research evolution, highlight subjects of relevance in that area, and determine potential gaps where further research is required. Evaluation of high-impact studies in specialties, subspecialties, journals, clinical topics, and research types has been a matter of interest that has received attention in recent years. However, analysis of high-impact AI studies remains a topic that is limited to a few publications [1-4].

Previous bibliometric analyses on AI have primarily focused on other medical specialties. Studies have examined the top 100 most cited articles in general medical AI, orthopedics, and radiology, as well as the most influential papers in medical AI across multiple disciplines [1-4]. These works collectively highlight how AI research has evolved and which themes attract the highest academic impact. However, none of these bibliometric studies have specifically explored highly cited AI-related publications within neurosurgical journals (NSJs), leaving a clear gap that this study aims to address.

To our knowledge, studies focusing on high-impact articles related to the utilization of AI in the field of neurosurgery are currently lacking in the literature. This study aimed to describe the characteristics of high-impact AI publications in the NSJs and to determine the factors that influence their citation numbers. Therefore, this study aimed to perform a comprehensive bibliometric analysis of highly cited AI publications in NSJs, describing their bibliometric characteristics and identifying factors that influence citation performance.

## Review

Methodology

Eligibility Criteria

This bibliometric study included articles published in predefined neurosurgical and spine journals (Table 1) that explicitly addressed AI or related technologies. Eligible articles were original research, review articles, technical notes, or case series describing or applying AI methods such as VR or AR, neuronavigation, robotics, ML, DL, ANNs, NLP, computer vision, or big-data analytics. Editorials, letters, commentaries, conference abstracts, corrigenda, and retractions were excluded. No language restrictions were applied, and all articles published up to the search date were considered. Articles were required to have ≥100 Google Scholar citations on a fixed date (October 15, 2024) to ensure inclusion of highly cited, impactful work.

**Table 1:** List of the searched neurosurgical and spine journals listed by the number of screened articles.

Searched journals	Number of screened articles	Number of selected high-impact articles
World Neurosurgery	264	5
Neurosurgery	147	23
European Spine Journal	88	1
Spine	78	7
Neurosurgical Focus	74	1
Journal of Neurosurgery	63	9
Spine Journal	60	5
Neurosurgical Review	40	2
Acta Neurochirurgica	40	2
Neurospine	38	1
Journal of Neurosurgery Spine	34	2
Clinical Neurology and Neurosurgery	29	1
Surgical Neurology International	22	2
Child's Nervous System	14	1
Journal of Neurosurgical Sciences	13	0
British Journal of Neurosurgery	11	1
Journal of Neurosurgery Pediatrics	10	0
Pituitary	10	0
Stereotactic and Functional Neurosurgery	9	3
Neurologia Medico-Chirurgica	9	0
Journal of Neurology, Neurosurgery, and Psychiatry	8	0
Joint Bone Spine	8	0
Journal of Korean Neurosurgical Society	7	0
Journal of Neurological Surgery Part A Central European Neurosurgery	7	0
Journal of Neurological Surgery Part B Skull Base	7	0
Surgical Neurology	5	0
Spinal Cord	5	0
Asian Journal of Neurosurgery	2	0
Pediatric Neurosurgery	3	0
Clinical Neurosurgery	1	0
Total	1,106	66

Information Sources

Data were retrieved from three publicly accessible sources. PubMed/MEDLINE was used to identify all eligible publications. Citation counts for each included article were extracted from Google Scholar on October 15, 2024, to capture a single-day snapshot and minimize citation drift. Journal-level metrics, including impact factors (IFs), were obtained from Bioxbio 2022 to maintain transparency of data provenance [20]. No other databases or commercial tools were used.

Search Strategy

Searches were conducted in PubMed from July 1 to 31, 2024, using a comprehensive Boolean query that combined AI-related terms with journal-specific filters. No date or language restrictions were applied. The AI concept block included terms such as “artificial intelligence,” “machine learning,” “deep learning,” “neural network,” “natural language processing,” “computer vision,” “virtual reality,” “augmented reality,” “robotics,” “data mining,” and “neuronavigation.” The journal block encompassed all indexed neurosurgical and spine journals relevant to the field.

The PubMed database was searched in July 2024 for suitable articles using the following combinations: [Title] “artificial intelligence” OR “AI” OR “artificial automation” OR “automated detection” OR “augmented reality” OR “ChatGPT” OR “computer vision” OR “computer assisted diagnosis” OR “convolutional neural network” OR “critical event detection” OR “data mining” OR “decision support” OR “digital assistant” OR “deep learning” OR “deep neural network” OR “fuzzy logic” OR “image analysis” OR “instrument detection” OR “machine learning” OR “natural language processing” OR “neural network” OR “neural networks” OR “phase detection” OR “recurrent neural network” OR “robotics” OR “text mining” OR “topic modelling” OR “virtual reality” OR “visual assistant”, AND [Journal] “individual neurosurgical and spine journals by name”. The list of neurosurgical and spine journals searched, and the number of screened articles, is shown in Table 1.

Selection Process

**Figure 1:**
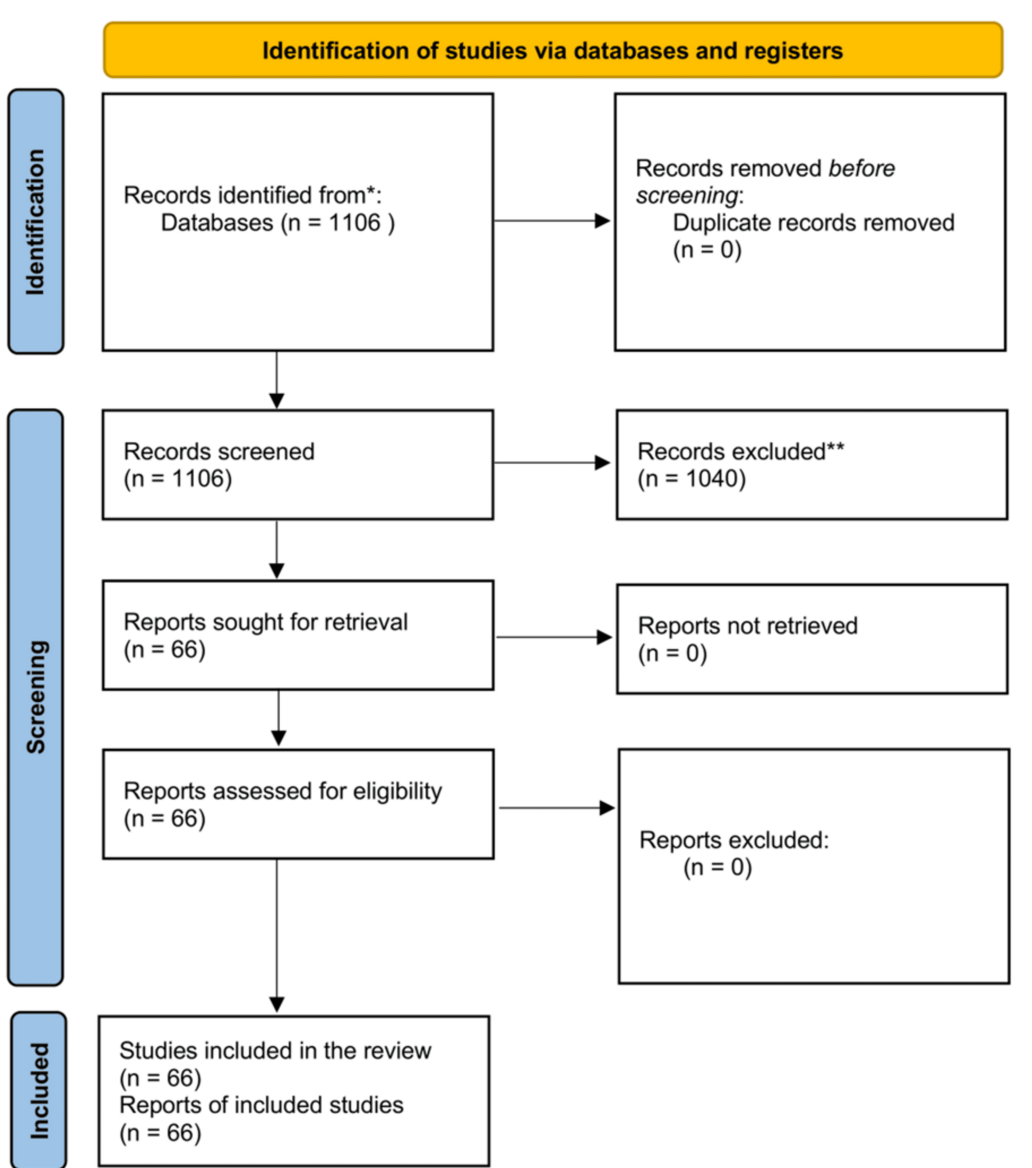
PRISMA flowchart of included studies. Source: [21]. PRISMA, Preferred Reporting Items for Systematic Reviews and Meta-Analyses

Two reviewers independently screened all PubMed results at the title and abstract level to determine eligibility. Articles meeting the journal and AI inclusion criteria were then assessed for citation counts using Google Scholar. Those with at least 100 citations as of October 15, 2024, were included in the analysis. Disagreements were resolved by consensus, and a third reviewer was available if needed. Reasons for exclusion included non-AI topic, wrong article type, publication outside the selected journals, or citation count below the predefined threshold. A Preferred Reporting Items for Systematic Reviews and Meta-Analyses (PRISMA) 2020 flow diagram summarizing the selection process was generated, with 1,106 records identified, 1,040 excluded, and 66 included in the final dataset (Figure 1) [21].

Data Collection Process

Two reviewers extracted data independently using a piloted spreadsheet to ensure accuracy and consistency. A random 10% sample was verified by a third reviewer to assess inter-rater reliability. Any discrepancies were resolved by consensus following review of the original publication. Data sources included PubMed metadata, journal websites, and Google Scholar citation pages.

Data Items

The following data were extracted for each included article: publication year, journal name, article type, first-author country, number of authors, number of centers or countries involved, and sample size (if applicable). Each study was categorized according to neurosurgical subspecialty (general neurosurgery, spine, neuro-oncology, education, cerebrovascular, endoscopy, or other) and type of AI technology (ML, DL, VR or AR, neuronavigation, robotics, NLP, or big-data analytics). Applications were further classified as surgical planning or assistance, training and education, diagnostic enhancement, predictive analytics, medical imaging, or personalized medicine. Citation metrics included total Google Scholar citations and the annual citation rate, defined as total citations divided by article age in years. Journal IF (Bioxbio 2022) was also recorded.

Study Risk-of-Bias Assessment

Traditional risk-of-bias tools were not applicable because this study analyzed bibliometric data rather than clinical outcomes. Instead, potential biases specific to bibliometric research were addressed. Coverage bias was minimized by including all PubMed-indexed neurosurgical and spine journals. Measurement bias related to citation variability was mitigated by capturing all citation data on the same day. Time-lag bias (favoring older articles) was addressed by calculating annual citation rates. Classification bias in subspecialty or technology coding was minimized through dual independent extraction and consensus review.

Synthesis Methods

Descriptive statistics summarized all extracted data. Continuous variables were reported as means ± standard deviations and medians (range), while categorical variables were presented as frequencies and percentages. Comparisons between groups were conducted using Welch’s two-sample t-test with a two-sided significance threshold of α = 0.05. Analyses were performed using MedCalc (Ostend, Belgium) [22]. No correction for multiple comparisons was applied, as analyses were exploratory.

Results

Study Selection and General Characteristics

A total of 66 high-impact AI research studies published in neurosurgical and spine journals were included (Table 2). The median (range) publication year and article age were 2015-2016 (1994-2013) and 8.5 (1-30) years, respectively. The median (range) IF of the publishing journals was 4.42 (0.76-5.53). The median (range) numbers of authors, centers, and countries per article were 6 (1-16), 2 (1-13), and 1 (1-4), respectively.

**Table 2:** The selected 66 high-impact AI publications in neurosurgical journals listed by their citation numbers. AI, artificial intelligence

No.	Articles	Total citations	Citations per year
1	Senders JT, et al. Machine learning and neurosurgical outcome prediction: a systematic review. World Neurosurg. 2018; 109: 476-486.e1 [23]	406	67.7
2	Meola A, et al. Augmented reality in neurosurgery: a systematic review. Neurosurg Rev. 2017; 40: 537-548. [24]	333	47.6
3	Bernardo A. Virtual reality and simulation in neurosurgical training. World Neurosurg. 2017; 106: 1015-1029 [25]	317	45.3
4	Roser F, et al. Spinal robotics: current applications and future perspectives. Neurosurgery. 2013; 72 Suppl 1: 12-8 [26]	307	27.9
5	Kim M, et al. Deep learning in medical imaging. Neurospine. 2019; 16: 657-668 [27]	302	60.4
6	Kockro RA, et al. Planning and simulation of neurosurgery in a virtual reality environment. Neurosurgery. 2000; 46: 118-35 [28]	299	74.8
7	Overley SC, et al. Navigation and robotics in spinal surgery: where are we now? Neurosurgery. 2017; 80: S86-S99. [29]	297	42.4
8	Lemole GM Jr, et al. Virtual reality in neurosurgical education: part-task ventriculostomy simulation with dynamic visual and haptic feedback. Neurosurgery. 2007; 61: 142-8 [30]	259	37
9	Nathoo N, et al. In touch with robotics: neurosurgery for the future. Neurosurgery. 2005; 56: 421-33 [31]	251	13.2
10	Kawamata T, et al. Endoscopic augmented reality navigation system for endonasal transsphenoidal surgery to treat pituitary tumors: technical note. Neurosurgery. 2002; 50: 1393-7 [32]	246	11.2
11	Senders JT, et al. Natural and artificial intelligence in neurosurgery: a systematic review. Neurosurgery. 2018; 83: 181-192 [33]	241	34.4
12	Alaraj A, et al. Virtual reality training in neurosurgery: Review of current status and future applications. Surg Neurol Int. 2011; 2: 52 [34]	236	18.2
13	Besharati Tabrizi L, Mahvash M. Augmented reality-guided neurosurgery: accuracy and intraoperative application of an image projection technique. J Neurosurg. 2015; 123: 206-11. [35]	231	25.7
14	Lee JC, et al. Quantitative analysis of back muscle degeneration in the patients with the degenerative lumbar flat back using a digital image analysis: comparison with the normal controls. Spine (Phila Pa 1976). 2008; 33: 318-25 [36]	209	13.1
15	Stadie AT, et al. Virtual reality system for planning minimally invasive neurosurgery. Technical note. J Neurosurg. 2008; 108: 382-94 [37]	204	12.8
16	Joseph JR, et al. Current applications of robotics in spine surgery: a systematic review of the literature. Neurosurg Focus. 2017; 42: E2 [38]	201	28.7
17	Alaraj A, et al. Virtual reality cerebral aneurysm clipping simulation with real-time haptic feedback. Neurosurgery. 2015; 11: 52-8 [39]	200	22.2
18	Buchlak QD, et al. Machine learning applications to clinical decision support in neurosurgery: an artificial intelligence augmented systematic review. Neurosurg Rev. 2020; 43: 1235-1253 [40]	199	49.8
19	Pfandler M, et al. Virtual reality-based simulators for spine surgery: a systematic review. Spine J. 2017; 17: 1352-1363 [41]	193	27.6
20	Belić M, et al. Artificial intelligence for assisting diagnostics and assessment of Parkinson's disease-A review. Clin Neurol Neurosurg. 2019; 184: 105442. [42]	187	37.4
21	Chan S, et al. Virtual reality simulation in neurosurgery: technologies and evolution. Neurosurgery. 2013; 72: 154-64. [43]	186	16.9
22	Elmi-Terander A, et al. Pedicle screw placement using augmented reality surgical navigation with intraoperative 3D imaging: a first in-human prospective cohort study. Spine (Phila Pa 1976). 2019; 44: 517-525. [44]	183	36.6
23	Ghasem A, et al. The arrival of robotics in spine surgery: a review of the literature. Spine (Phila Pa 1976). 2018; 43: 1670-1677 [45]	180	30
24	Alaraj A, et al. Role of cranial and spinal virtual and augmented reality simulation using immersive touch modules in neurosurgical training. Neurosurgery. 2013; 72: 115-23. [46]	180	16.4
25	Kochanski RB et al. Image-guided navigation and robotics in spine surgery. Neurosurgery. 2019; 84: 1179-1189 [47]	180	36
26	Ali R, et al. Performance of ChatGPT, GPT-4, and Google Bard on a neurosurgery oral boards preparation question bank. Neurosurgery. 2023; 93: 1090-1098 [48]	175	175
27	Louw DF, et al. Surgical robotics: a review and neurosurgical prototype development. Neurosurgery. 2004; 54: 525-36 [49]	168	8.4
28	Abe Y, et al. A novel 3D guidance system using augmented reality for percutaneous vertebroplasty: technical note. J Neurosurg Spine. 2013; 19: 492-501 [50]	168	15.3
29	Kim JS, et al. Examining the ability of artificial neural networks machine learning models to accurately predict complications following posterior lumbar spine fusion. Spine (Phila Pa 1976). 2018; 43: 853-860 [51]	167	27.8
30	Davis MC, et al. Virtual interactive presence in global surgical education: international collaboration through augmented reality. World Neurosurg. 2016; 86: 103-11 [52]	166	20.8
31	de Faria JW, et al. Virtual and stereoscopic anatomy: when virtual reality meets medical education. J Neurosurg. 2016; 125: 1105-1111. [53]	163	20.4
32	Spicer MA, Apuzzo ML. Virtual reality surgery: neurosurgery and the contemporary landscape. Neurosurgery. 2003; 52: 489-97 [54]	163	7.8
33	Choudhury N, et al. Fundamentals of neurosurgery: virtual reality tasks for training and evaluation of technical skills. World Neurosurg. 2013; 80: e9-1 [55]	162	14.7
34	Senders JT, et al. An introduction and overview of machine learning in neurosurgical care. Acta Neurochir (Wien) 2018; 160: 29-38 [56]	160	26.7
35	Shenai MB, et al. Virtual interactive presence and augmented reality (VIPAR) for remote surgical assistance. Neurosurgery. 2011; 68: 200-7 [57]	160	12.3
36	Henn JS, et al. Interactive stereoscopic virtual reality: a new tool for neurosurgical education. Technical note. J Neurosurg. 2002; 96: 144-9 [58]	160	7.3
37	Elmi-Terander A, et al. Surgical navigation technology based on augmented reality and integrated 3D intraoperative imaging: a spine cadaveric feasibility and accuracy study. Spine (Phila Pa 1976). 2016; 41: E1303-E1311 [59]	151	18.9
38	Cabrilo I, et al. Augmented reality in the surgery of cerebral aneurysms: a technical report. Neurosurgery. 2014; 10: 252-60 [60]	149	14.9
39	Wesseling P, et al. Quantitative immunohistological analysis of the microvasculature in untreated human glioblastoma multiforme. Computer-assisted image analysis of whole-tumor sections. J Neurosurg. 1994; 81: 902-9 [61]	142	4.7
40	Kockro RA, et al. A collaborative virtual reality environment for neurosurgical planning and training. Neurosurgery. 2007; 61: 379-91 [62]	136	8
41	Karhade AV, et al. Development of machine learning algorithms for prediction of 30-day mortality after surgery for spinal metastasis. Neurosurgery. 2019; 85: E83-E91 [63]	136	27.2
42	Luciano CJ, et al. Learning retention of thoracic pedicle screw placement using a high-resolution augmented reality simulator with haptic feedback. Neurosurgery. 2011; 69: ons14-9 [64]	132	10.2
43	Galbusera F, et al. Fully automated radiological analysis of spinal disorders and deformities: a deep learning approach. Eur Spine J. 2019; 28: 951-960 [65]	126	25.2
44	Ghaednia H, et al. Augmented and virtual reality in spine surgery, current applications and future potentials. Spine J. 2021; 21: 1617-1625 [66]	125	41.7
45	Elmi-Terander A, et al. Feasibility and accuracy of thoracolumbar minimally invasive pedicle screw placement with augmented reality navigation technology. Spine (Phila Pa 1976). 2018; 43: 1018-1023 [67]	125	20.8
46	Rughani AI, et al. Use of an artificial neural network to predict head injury outcome. J Neurosurg. 2010; 113: 585-90 [68]	123	8.8
47	Low D, et al. Augmented reality neurosurgical planning and navigation for surgical excision of parasagittal, falcine and convexity meningiomas. Br J Neurosurg. 2010; 24: 69-74 [69]	122	8.7
48	Banerjee PP, et al. Accuracy of ventriculostomy catheter placement using a head- and hand-tracked high-resolution virtual reality simulator with haptic feedback. J Neurosurg. 2007; 107: 515-21 [70]	121	7.1
49	Cabrilo I, et al. Augmented reality in the surgery of cerebral arteriovenous malformations: technique assessment and considerations. Acta Neurochir (Wien). 2014; 156: 1769-74 [71]	120	12
50	Chang SD, Adler JR. Robotics and radiosurgery--the cyberknife. Stereotact Funct Neurosurg. 2001; 76: 204-8 [72]	120	5.2
51	Molina CA, et al. Augmented reality-assisted pedicle screw insertion: a cadaveric proof-of-concept study. J Neurosurg Spine. 2019; 31: 139-146 [73]	119	23.8
52	Gleason PL, et al. Video registration virtual reality for nonlinkage stereotactic surgery. Stereotact Funct Neurosurg. 1994; 63: 139-43 [74]	116	3.9
53	Robison RA, et al. Man, mind, and machine: the past and future of virtual reality simulation in neurologic surgery. World Neurosurg. 2011; 76: 419-30. [75]	116	8.9
54	Wong GK, et al. Craniotomy and clipping of intracranial aneurysm in a stereoscopic virtual reality environment. Neurosurgery. 2007; 61: 564-8 [76]	115	6.8
55	Ames CP, et al. Artificial intelligence based hierarchical clustering of patient types and intervention categories in adult spinal deformity surgery: towards a new classification scheme that predicts quality and value. Spine (Phila Pa 1976). 2019; 44: 915-926 [77]	113	22.6
56	Deng W, et al. Easy-to-use augmented reality neuronavigation using a wireless tablet PC. Stereotact Funct Neurosurg. 2014; 92: 17-24 [78]	110	11
57	Shi HY, et al. In-hospital mortality after traumatic brain injury surgery: a nationwide population-based comparison of mortality predictors used in artificial neural network and logistic regression models. J Neurosurg. 2013; 118: 746-52 [79]	109	9.9
58	Karhade AV, et al. Machine learning for prediction of sustained opioid prescription after anterior cervical discectomy and fusion. Spine J. 2019; 19: 976-983 [80]	108	21.6
59	Godil SS, et al. Fuzzy logic: A "simple" solution for complexities in neurosciences? Surg Neurol Int. 2011; 2: 24 [81]	107	8.2
60	Ali R, et al. Performance of ChatGPT and GPT-4 on neurosurgery written board examinations. Neurosurgery. 2023; 93: 1353-1365 [82]	107	107
61	Müller F, et al. Augmented reality navigation for spinal pedicle screw instrumentation using intraoperative 3D imaging. Spine J. 2020; 20: 621-628 [83]	106	26.5
62	Arle JE, et al. Prediction of posterior fossa tumor type in children by means of magnetic resonance image properties, spectroscopy, and neural networks. J Neurosurg. 1997; 86: 755-61 [84]	105	3.9
63	Kockro RA, et al. Dex-ray: augmented reality neurosurgical navigation with a handheld video probe. Neurosurgery. 2009; 65: 795-807 [85]	105	7
64	Karhade AV, et al. Development of machine learning algorithms for prediction of prolonged opioid prescription after surgery for lumbar disc herniation. Spine J. 2019; 19: 1764-1771 [86]	103	20.6
65	Cohen AR, et al. Virtual reality simulation: basic concepts and use in endoscopic neurosurgery training. Childs Nerv Syst. 2013; 29(8): 1235-44 [87]	100	9.1
66	Senders JT, et al. An Online Calculator for the prediction of survival in glioblastoma patients using classical statistics and machine learning. Neurosurgery. 2020; 86: E184-E192 [88]	100	25

Thirty-one (47%) articles reported a sample size, with a median (range) of 33 (3-22,629) participants. The median (range) total citation count was 173 (100-406), and the median annual citation rate was 20.6 (3.9-175) citations per year. The top five articles by annual citation rate are summarized in Table 3.

**Table 3:** The top 5 of the selected 66 high-impact AI publications in neurosurgical journals, listed by citations per year. AI, artificial intelligence

Articles	Citations per year	Total citations	Rank by total citations
Ali R, et al. Performance of ChatGPT, GPT-4, and Google Bard on a neurosurgery oral boards preparation question bank. Neurosurgery. 2023; 93(5): 1090-1098 [48]	175	175	26
Ali R, et al. Performance of ChatGPT and GPT-4 on neurosurgery written board examinations. Neurosurgery. 2023; 93(6): 1353-1365 [82]	107	107	60
Kockro RA, et al. Planning and simulation of neurosurgery in a virtual reality environment. Neurosurgery. 2000; 46(1): 118-35 [28]	74.8	299	6
Senders JT, et al. Machine learning and neurosurgical outcome prediction: a systematic review. World Neurosurg. 2018; 109: 476-486.e1 [27]	67.7	406	1
Kim M, et al. Deep learning in medical imaging. Neurospine. 2019; 16(4): 657-668 [23]	60.4	302	5

Citation Analysis by Article Age and Study Features

Newer studies published in the last decade had significantly higher mean citation numbers compared with older studies (≥10 years) (189.9 vs. 154.6; *P* = 0.033).

Among these, 14 of 34 (41%) newer studies were review articles, compared to 9 of 32 (28%) older ones. Citation rates were not significantly affected by sample size (<33 vs. ≥33), journal IF (<4.42 vs. ≥4.42), number of authors (<6 vs. ≥6), centers (1 vs. >1), or countries (1 vs. >1). Detailed analyses are provided in Table 4.

**Table 4:** Citation analysis for the selected 66 high-impact AI publications in neurosurgical journals according to several related parameters. *Significant at *P* < 0.05. **Reported in 31 studies only AI, artificial intelligence; No., number; SD, standard deviation; CI, confidence interval

Feature	Variables	No. of articles, *n* (%)	Mean citations (±SD)	Mean difference (95% CI)	*t*-test	*P*-value
Article’s age	≥10 years	32 (48%)	154.6 (±51)	35.3 (2.9-67.7)	2.20	0.033*
	<10 years	34 (52%)	189.9 (±77.1)			
Journal impact factor	≥4.42	32 (48%)	176.6 (±61.1)	-7.3 (-40.8 to 26.2)	0.44	0.6647
	<4.42	34 (52%)	169.3 (±74)			
Number of authors	≥6	39 (59%)	166.3 (±70.2)	15.9 (-17.9 to 49.7)	0.94	0.3515
	<6	27 (41%)	182.2 (±63.8)			
Number of centers	1	32 (48%)	170.5 (±61.9)	4.5 (-29 to 38)	0.27	0.7893
	>1	34 (42%)	175 (±73.4)			
Number of countries	1	46 (70%)	177.4 (±61.6)	15.1 (-51.4 to 21.2)	0.84	0.4085
	>1	20 (30%)	162.3 (±80.5)			
Sample size**	≥33	16 (24%)	142.4 (±52.9)	25.8 (114.8-66.4)	1.30	0.2038
	<33	15 (23%)	168.2 (±57.6)			

Journal Distribution and Citation Impact

The most frequent publishing journals were Neurosurgery (23 articles, 35%), Journal of Neurosurgery (9 articles, 14%), Spine (7 articles, 11%), World Neurosurgery (5 articles, 8%), and Spine Journal (5 articles, 8%). The full journal citation analysis is summarized in Table 5. Articles published in World Neurosurgery demonstrated significantly higher mean citation numbers than those published in other journals (233.4 vs. 167.9, *P* = 0.0361). Notably, three out of the five World Neurosurgery articles (60%) were review articles, suggesting that review papers in this journal may be associated with stronger citation performance (Table 5).

**Table 5:** Citation analysis for the selected 66 high-impact AI publications in neurosurgical journals according to the publishing journal. *Significant at *P* < 0.05. **Others (articles): Journal of Neurosurgery Spine (2), Neurosurgical Review (2), Surgical Neurology International (2), Acta Neurochirurgica (2), British Journal of Neurosurgery (1), European Spine Journal (1), Neurosurgical Focus (1), Clinical Neurology and Neurosurgery (1), Neurospine (1), and Child's Nervous System (1). AI, artificial intelligence; No., number; SD, standard deviation; CI, confidence interval

Journal	No. of articles (%) (journal)	Mean citations (±SD) (journal)	No. of articles (%) (other journals)	Mean citations (±SD) (other journals)	Mean difference (95% CI)	*t*- Test	*P*-value
Neurosurgery	23 (35%)	186.6 (±64.8)	43(65%)	165.4 (±68.7)	-21.2 (-56 to 13.6)	1.22	0.2277
Journal of Neurosurgery	9 (14%)	150.9 (±43.4)	57 (86%)	176.3 (±70.3)	25.4 (-23 to 73.8)	1.05	0.2983
Spine	7 (11%)	161.1 (±33.9)	59 (89%)	174 (±70.6)	13.1 (-41.2 to 67.4)	0.49	0.6315
World Neurosurgery	5 (8%)	233.4 (±122.7)	61 (92%)	167.9 (±60.1)	-65.5 (-126.6 to -4.4)	2.15	0.0361*
Spine Journal	5 (8%)	127 (±37.9)	61 (92%)	176.6 (±68.3)	49.6 (-12.5 to 111.7)	1.60	0.1154
Stereotactic and Functional Neurosurgery	3 (5%)	115.3 (±5)	63 (95%)	175.6 (±68)	60.3 (-18.7 to 139.3)	1.52	0.1323
Others**	14 (21%)	177.1 (±72.3)	52 (79%)	171.7 (±67)	-5.4 (-46.4 to 35.6)	0.26	0.7932

First Author Country and Citation Patterns

The most common first-author countries were the United States (34 studies, 52%), Germany (5 studies, 8%), the Netherlands (4 studies, 6%), and Switzerland (4 studies, 6%). The country-based citation analysis is provided in Table 6. Articles led by first authors from the Netherlands had significantly higher mean citation numbers compared with those from other countries (237.3 vs. 168.7, *P* = 0.0484). Of the four studies first authored by researchers from the Netherlands, three (75%) were review articles, indicating a possible association between review-type publications and high citation performance within this subgroup (Table 6).

**Table 6:** Citation analysis for the selected 66 high-impact AI publications in neurosurgical journals according to the first author’s country. *Significant at *P* < 0.05. **Others (articles): Korea (2), Singapore (2), Japan (2), Canada (2), Australia (1), Serbia (1), Brazil (1), Italy (1), and Pakistan (1). AI, artificial intelligence; No., number; SD, standard deviation; CI, confidence interval

Country	No. of articles (%) (country)	Mean citations (±SD) (country)	No. of articles (%) (other countries)	Mean citations (±SD) (other countries)	Mean difference (95% CI)	*t*-test	*P*-value
United States	34 (52%)	166.3 (±64)	32 (48%)	179.7 (±71.6)	13.4 (-20 to 46.8)	0.81	0.4252
Germany	5 (8%)	214.2 (±62.4)	61 (92%)	169.4 (±67.4)	-44.8 (-107.2 to 17.6)	1.45	0.1561
The Netherlands	4 (6%)	237.3 (±120.5)	62 (94%)	168.7 (±62.2)	-68.8 (-136.7 to 0.49)	2.02	0.0484*
Switzerland	4 (6%)	120 (±20.5)	62 (94%)	176.2 (±68.2)	56.2 (-12.6 to 125)	1.64	0.1075
Sweden	3 (5%)	153 (±29.1)	63 (95%)	173.8 (±68.9)	20.8 (-59.5 to 101.1)	0.52	0.6066
China	3 (5%)	111.3 (±3.2)	63 (95%)	175.8 (±67.8)	64.5 (-14.3 to 143.3)	1.65	0.1068
Others**	13 (20%)		53 (80%)	168.8 (±68.9)	-20.3 (-62.1 to 21.5)	0.97	0.3360

Study Design, Distribution, and Citation Analysis

The study designs of the included articles were classified as technical notes (26 studies, 39%), review articles (23 studies, 35%), original research (14 studies, 21%), and case series (3 studies, 5%). As summarized in Table 7, review articles demonstrated significantly higher mean citation numbers compared with other study designs (209.9 vs. 153, *P* = 0.0008), while original research articles had significantly lower mean citation numbers (127.4 vs. 185.1, *P* = 0.0038). More than half of the review articles (13 of 23; 57%) focused on general neurosurgery topics, compared with only two of the fourteen original research papers (14%). Although few in number, case series studies showed significantly higher mean citation counts compared to other designs (249 vs. 169.2, *P* = 0.0448). In contrast, technical notes did not demonstrate a statistically significant difference in citation impact (Table 7).

**Table 7:** Citation analysis for the selected 66 high-impact AI publications in neurosurgical journals according to the study design. *Significant at *P* < 0.05. AI, artificial intelligence; No., number; SD, standard deviation; CI, confidence interval

Study design	No. of articles (%) (design)	Mean citations (±SD) (design)	No. of articles (%) (other designs)	Mean citations (±SD) (other designs)	Mean difference (95% CI)	*t*-test	*P*-value
Technical note	26 (39%)	155.7 (±49.8)	40 (61%)	184 (±75.5)	28.3 9-5.2 to 61.8)	1.70	0.0968
Review	23 (35%)	209.9 (±78)	43 (65%)	153 (±52.2)	-56.9 (-89 to 24.8)	3.45	0.0008*
Original research	14 (21%)	127.4 (±28.1)	52 (79%)	185.1 (±70)	57.7 (19.3 to 96.1)	3.13	0.0038*
Case series	3 (5%)	249 (±51.4)	63 (95%)	169.2 (±66.4)	-79.8[ (-157.7) to (-1.9)]	2.06	0.0448*

Subspecialty Trends and Citation Outcomes

Among the included studies, the most common subspecialties were general neurosurgery (21 studies, 32%), spine (21 studies, 32%), education (6 studies, 9%), and neuro-oncology (6 studies, 9%) (Table 8). Articles classified under general neurosurgery were associated with significantly higher mean citation numbers than those in other subspecialties (197.3 vs. 161.4, *P* = 0.0475). Of the 21, 14 (67%) general neurosurgery studies were review articles, compared with nine of the 45 (20%) studies from other subspecialties, reinforcing the association between review design and higher citation performance within this domain (Table 8).

**Table 8:** Citation analysis for the selected 66 high-impact AI publications in neurosurgical journals according to the study subspecialty. *Significant at *P* < 0.05. **Others (articles): Trauma (2), Functional neurosurgery (1), Skull Base (1), Radiosurgery (1), Pediatric Neurosurgery (1). AI, artificial intelligence; No., number; SD, standard deviation; CI, confidence interval

Subspecialty	No. of articles (%) (subspecialty)	Mean citations (±SD) (subspecialty)	No. of articles (%) (other subspecialties)	Mean citations (±SD) (other subspecialties)	Mean difference (95% CI)	*t*-test	*P*-value
General neurosurgery	21 (32%)	197.3 (±84.3)	45 (68%)	161.4 (±55.7)	-35.9 (-70.7 to -1.1)	2.01	0.0475*
Spine	21 (32%)	163.3 (±56.8)	45 (68%)	177.3 (±72.2)	14 (-21.8 to 49.8)	0.79	0.4372
Education	6 (9%)	181.5 (±46)	60 (91%)	172 (±69.6)	-9.4 (-67.7 to 48.7)	0.33	0.7455
Neurooncology	6 (9%)	144.5 (±47.9)	60 (91%)	175.7 (±68.9)	31.2 (-26.5 to 88.9)	1.09	0.2844
Endoscopic surgery	3 (5%)	183.3 (±75.2)	63 (95%)	172.3 (±67.8)	-11 (-91.3 to 69.3)	0.27	0.7853
Cerebrovascular	3 (5%)	128 (±18.4)	63 (95%)	175 (±68.4)	47 (-32.6 to 126.6)	1.19	0.2424
Others^**^	6 (9%)	177.8 (±88.3)	60 (91%)	174.4 (±67.2)	-3.4 (-62.5 to 55.7)	0.11	0.9088

AI Technology Categories

The predominant AI technologies used across the included articles were VR (19 studies, 29%), ML (13 studies, 20%), AR (10 studies, 15%), neuronavigation (10 studies, 15%), and robotic-assisted systems (6 studies, 9%). A detailed analysis of citation performance by technology category is provided in Table 9. No statistically significant differences in citation numbers were identified among the different AI technology types, indicating that citation performance was not dependent on the specific technology applied (Table 9).

**Table 9:** Citation analysis for the selected 66 high-impact AI publications in neurosurgical journals according to the utilized AI technology. *Others (articles): Deep learning (2), Natural language processing (2), and big-data analytics (1). AI, artificial intelligence; No., number; CI, confidence interval

Technology	No. of articles (%) (technology)	Mean citations (±SD) (technology)	No. of articles (other technologies)	Mean citations (±SD) (other technologies)	Mean difference (95% CI)	*t*-test	*P*-value
Virtual reality	19 (29%)	170.8 (±56.3)	47 (71%)	173.6 (72.2)	2.8 (-34.2 to 39.8)	0.15	0.8803
Machine learning	13 (20%)	169.9 (±84.7)	53 (80%)	173.5 (63.7)	3.6 (-38.5 to 45.7)	0.17	0.8650
Augmented reality	10 (15%)	175 (±65.8)	56 (85%)	172.4 (68.5)	-2.6 (-49.3 to 44.1)	0.11	0.9118
Neuronavigation	10 (15%)	175.8 (±76.5)	56 (85%)	172.3 (66.6)	-3.5 (-50.2 to 43.2)	0.15	0.8814
Robotic-assisted systems	6 (9%)	204.5 (±66)	60 (91%)	169.7 (67.5)	-34.8 (-92.4 to 22.8)	1.21	0.2322
Artificial neural network	3 (5%)	133 (±30.3)	63 (95%)	174.7 (68.4)	41.7 (-38 to 121.4)	1.05	0.3000
Others*	5 (8%)	163.4 (±82.3)	61 (92%)	173.6 (67)	10.2 (-53 to 73.4)	0.28	0.7842

AI Applications and Citation Outcomes

The main applications of AI in the analyzed studies were surgical planning and assistance (33 studies, 50%), training and education (12 studies, 18%), outcome prediction models (8 studies, 12%), predictive analytics (6 studies, 9%), and medical imaging (4 studies, 6%) (Table 10). Analysis of citation metrics by application type revealed no statistically significant differences in citation performance across these categories. This suggests that the impact and visibility of highly cited AI research in neurosurgery are driven more by study design and topic breadth than by the specific AI application focus (Table 10).

**Table 10:** Citation analysis for the selected 66 high-impact AI publications in neurosurgical journals according to the utilized AI application. *Others (articles): Diagnostic enhancement (2), Personalized medicine (1). AI, artificial intelligence; No., number; CI, confidence interval

Application	No. of articles (%) (application)	Mean citations (±SD) (application)	No. of articles (other applications)	Mean citations (±SD) (other applications)	Mean difference (95% CI)	*t*-test	*P*-value
Surgical planning and assistance	33 (50%)	183.9 (±68.4)	33 (50%)	161.7 (±65.9)	-22.2 (-55.2 to 10.8)	1.36	0.1841
Training	12 (18%)	159.3 (±43.4)	54 (82%)	175.8 (±71.9)	16.5 (-26.8 to 59.8)	0.76	0.4489
Outcome prediction models	8 (12%)	156.5 (±103.2)	58 (88%)	175.1 (±62.1)	18.6 (-32.5 to 69.7)	0.73	0.4698
Predictive analytics	6 (9%)	144.8 (±53.6)	60 (91%)	175.6 (±68.6)	30.8 (-27 to 88.6)	1.07	0.2909
Medical imaging	4 (6%)	189.5 (±86.5)	62 (94%)	171.7 (±66.9)	-17.8 (-87.8 to 52.2)	0.51	0.6133
Others*	3 (5%)	182 (±20)	63 (955)	172.4 ± (69.1)	-9.6 (-90 to 70.8)	0.24	0.8122

Discussion

AI in neurosurgery is an evolving and interdisciplinary field that attracts attention from neurosurgeons, scientists, and healthcare policymakers. Researchers from diverse backgrounds are likely to cite quality AI-related studies. The number of citations an article receives depends on multiple factors relating to the article, journal, authors, specialty, and trending topics [19].

The median article citation number for high-impact AI publications in NSJs was 173. This was lower than the 238 and 353 reported as the median citation numbers for the top 100 AI articles in radiology and medicine, respectively [1,4]. It was higher than the 51 stated as the median citation number for the top 100 AI studies in orthopedics [2]. It was also higher than the values of 52, 111, and 137 calculated as the median citation numbers for high-impact publications in NSJs that focused on other topics, such as bibliometric research, survey questionnaires, and Patient-Reported Outcome Measures, respectively [18,89,90]. The median annual citation rate was 20.6, which is comparable to that reported for the top 100 articles in medical AI [1]. Of interest that the two articles that had the highest annual citation rates (175 and 107 cites per year) addressed the performance of ChatGPT and GPT-4 in the neurosurgery oral and written board examination (Table 3), a reflection of the high relevance and potential impact of AI on neurosurgical training.

The median publication years of the articles were 2015-2016, which supports observations by others that most highly cited AI studies in medicine have been published during the past two decades [1,2,4]. In this study, review articles significantly impacted citation numbers. Publications within the last 10 years were also associated with significantly higher citation rates. The latter finding may have been influenced by the difference in the proportion of review articles between newer and older publications (41% vs. 28%). It is also recognized that the number of citations typically increases during the first year after publication, reaches a peak, and then declines as time passes [19].

Hence, a maximal citation number within the first 10 years after publication is not uncommon.

In this study, the highest number of articles was published in Neurosurgery (23 articles, 35%). The dominance of one journal in high-impact AI publications in the medical fields was reported in 8% to 38% of studies [1-4]. Journal IF differs considerably between the various specialties. The publishing journal’s IF ranged from 0.76 to 5.53. Zahoor et al. [3] reported a wider range of journal IF (0.94-87.24) for the 100 most influential papers in medical AI. Furthermore, and similar to other topics published in NSJs, we found that the citation numbers for AI publications in NSJs were not significantly impacted by the journal IF [18,89]. Surprisingly, articles published in World Neurosurgery were associated with significantly higher mean citation numbers. This could be because the number of such articles was small, and three of the five studies were review articles.

Sample size was reported in 47% of studies, and it did not significantly impact citation numbers. The influence of sample size on citation rates appears to vary by topic, with some publications in NSJs reporting a significant association [90-92], while others found none [18,23]. The most frequent first author’s country was the United States (52%). The dominance of the United States as the leading contributor to AI-related research is well recognized and has been documented in 36%-61% of high-impact publications across several medical fields [1-4]. Only articles published by first authors from the Netherlands were associated with significantly higher mean citation numbers compared to others. However, the number of articles was small [4], and all the studies were review articles. Furthermore, the number of authors, centers, and countries did not impact citation numbers, which is similar to the findings of other research topics published in NSJs [18,89,90,91,92].

The most common study designs were technical notes (26, 39%) and review articles (23, 35%). This could reflect the reliance of neurosurgery on new technologies that are reported in the literature as technical notes. The latter had no significant impact on citation numbers. However, significantly higher mean citation numbers were observed with review articles. This is not surprising as review articles are comprehensive, interdisciplinary, and have a broad appeal and are accessible to a wider audience. Original research accounted for 21% of studies, which is lower than the reported rates for original articles in the most cited studies of other medical fields [1,2,4]. Case series and original research articles had significantly higher and lower citation numbers than other article types, respectively, but their citation patterns may have been biased by their relatively small numbers (3 case series and 14 original research studies).

Different medical fields have specific areas where AI is more widely adopted [1-4]. The most common AI-related subspecialties of high-impact studies published in NSJs were general neurosurgery (21, 32%) and spine (21, 32%). This is not surprising, as general studies have a wider appeal, and the spine is heavily linked to AI technology due to high imaging volume, structured pathology, and AI applicability in diagnosis and surgical planning. Only articles that were considered general in their subspecialty were associated with a significantly higher mean citation number compared to others. This is not unusual as general articles attract higher citation rates because they appeal to a wider audience, serve as foundational references, remain relevant across multiple disciplines, and receive more visibility [19].

The integration of AI technology in high-impact publications varies significantly depending on the specialty in studied. In this study, the AI technologies most frequently used are well-documented in the literature. These included VR (29%) [5,6,10], ML (20%) [13,15,16], AR (15%) [5,6,15], neuro-navigation (15%) [5,15], and robotic-assisted systems (9%) [5,14]. Other reports of highly cited AI-related publications in medical specialties stated the most frequently used tools were ML (18%-26%) [1,2], DL (16%-34%) [2,4], NLP (20%) [1], convolutional neural networks (21%) [4], and ANNs (13%) [1]. The selection of technologies is related to needs specific needs of the specialty and underscores how AI is tailored to address unique challenges and data structures inherent to each discipline. Robotic-assisted systems were associated with the highest citation rates, but without reaching significance. The applications in most AI-related studies published in NSJs were surgical planning and assistance (50%) and education and training (18%). This is expected as the use of AI in neurosurgery differs from other medical specialties due to the high degree of precision requirement, real-time intraoperative integration, and advanced imaging analysis [9-11]. Turgut et al. [2] reported that 85% of the most cited AI studies related to orthopedics were diagnostic in nature. Surgical planning and assistance were associated with higher citation rates than the other applications, but the difference did not reach statistical significance.

There are several limitations to this study. The study relied on the precision of online search engines PubMed and Google Scholar. The study did not include AI research studies that were published in the NSJs. The selection of the 66 most cited studies was based on their total citations at a certain point, which was likely to change relatively quickly. This could have influenced the inclusion or exclusion of a few of the lower-impact bibliometric studies. The wide duration from publication (17 years) had probably affected the citations of older studies. The quality of the AI studies was not examined. Additionally, changing trends in the reporting of AI data over the years were not addressed. Errors may have occurred during data collection, and discrepancies may exist in the allocation of articles into various subspecialty, technology, and application categories. Defining affiliation based on the first author may not accurately reflect the contributions of all authors on multidisciplinary papers.

## Conclusions

This paper provides an insight into the areas of the most influential AI research work in NSJs. The neurosurgical literature on AI is expanding and increasing in influence. High-impact publications were predominantly review articles, focused on general neurosurgery, and primarily addressed surgical planning, assistance, and education. AI will continue to shape the future of neurosurgery; more original research work is required to translate the AI advancements into routine neurosurgical applications.
